# A novel form of wasp mimicry in a new species of praying mantis from the Amazon rainforest, *Vespamantoida wherleyi* gen. nov. sp. nov. (Mantodea, Mantoididae)

**DOI:** 10.7717/peerj.7886

**Published:** 2019-10-17

**Authors:** Gavin J. Svenson, Henrique M. Rodrigues

**Affiliations:** 1 Department of Invertebrate Zoology, Cleveland Museum of Natural History, Cleveland, OH, USA; 2 Department of Biology, Case Western Reserve University, Cleveland, OH, USA

**Keywords:** Hymenoptera, Mantoida, Neotropics, Praying mantis, Mimicry, Wasp, Morphology, Mantodea

## Abstract

A wasp mimicking praying mantis (Mantodea) of the early evolving Mantoididae family was discovered in 2013 at a research station near the Amazon River in Northern Peru. This adult specimen exhibited a striking bright red/orange and black coloration pattern that was undocumented in all known praying mantis species. We tested the status of this new specimen using external morphology, male genital dissections, and geographic distribution. Our findings demonstrate the specimen to represent a new species, *Vespamantoida wherleyi* gen. nov. sp. nov., that is closely allied with a recently described species, *Mantoida toulgoeti* Roy, 2010, both of which are included within the newly erected genus. To support our actions, we present high resolution images of museum preserved and living specimens, morphological illustrations, a generic-level distribution map, and recorded video of the behavior of the holotype taken in the field at the time of collection. The bright red/orange coloration contrasted with black markings, the general appearance of a hymenopteran that includes a narrowed wasp waist, and the locomotory patterns and antennal movements mark this newly discovered species as unique among all hymenopteran mimicking Mantoididae as well as all other praying mantises.

## Introduction

Much of the known diversity within Mantoididae, a small, early evolved lineage of praying mantises, is thought to mimic various types Hymenoptera, both morphologically and behaviorally (Jackson & Drummond, 1974; Deyrup, 1986; Agudelo, 2014). In fact, early instars of *Mantoida maya* Saussure & Zehntner, 1894 are highly similar to co-occurring ants of the genus *Camponotus*, while late instars of *Mantoida maya* begin to look like sympatric Vespidae wasps as they outgrow the general size of *Camponotus* during post-embryonic development (Jackson & Drummond, 1974). Further, the abdomen of *Mantoida maya* is ovoid with a posterior expansion that is coupled with narrowing anterior segments, which morphologically resemble a hymenopteran abdomen (Jackson & Drummond, 1974; Deyrup, 1986). Additionally, the coloration of the predominantly black abdomen is adorned with white, contrasting stripes that convincingly create the illusion of a petiole (Jackson & Drummond, 1974; Deyrup, 1986). The behavioral characteristics of *Mantoida maya* enhances their physical mimicry through hymenopteran-like locomotion, which includes rapid, jerky movements with sudden starts and stops, rapid antennal movements, and the repeated up-and-down pumping of the abdomen (Jackson & Drummond, 1974). Besides *Mantoida maya*, Agudelo (2014) remarked that the newly described *Paramantoida amazonica* Agudelo, 2014 was highly similar to aggressive wasps that share its size and contrasting black and white coloration.

Although hymenopteran mimicry has been documented in a number of other mantodean lineages, it is primarily restricted to early nymphal instars resembling ants that includes many species of Hymenopodidae (Kumar, 1973; Edmunds, 1976; Svenson et al., 2015), certain Paraoxypilinae (Milledge, 1990; Wieland, 2013), Acontistidae (Rivera & Svenson, 2016; Salazar, 2003), *Miomantis paykullii* Stål, 1871 (Edmunds, 1972; Kumar, 1973), *Miomantis aurea* Giglio-Tos, 1917 (Edmunds, 1976), *Polyspilota aeruginosa* Goeze, 1778 (Kumar, 1973), *Prohierodula ornatipennis* Bolivar, 1893 (Edmunds, 1976), *Mantillica nigricans* Westwood, 1889 (Agudelo & Rafael, 2014), *Ligaria senegalensis* Roy, 1961 (Gillon & Roy, 1968), *Gonypetella ìvoirensis* Gillon & Roy, 1969 (Gillon & Roy, 1968), *Tarachodes afzelii* Stål, 1871 (Edmunds, 1972), and *Sphodromantis lineola* (Edmunds, 1972). The diversity of lineages adopting this nymphal strategy is surprising and may indicate a deeper evolutionary strategy within the order. However, mimicry of hymenopterans in adult praying mantises is far less known and has only been suggested, outside of Mantoididae, in a few groups. Of these, some are mimics of ants, which include *Nesoxypilus* Beier, 1965 (Milledge, 1990), *Myrmecomantis* Giglio-Tos, 1913 (Milledge, 1990), and *Mantillica nigricans* (Agudelo & Rafael, 2014). Although the name would suggest mimicry of the beetle genus *Tricondyla* Latreille, 1822, the Southeast Asian mantis genus *Tricondylomimus* Chopard, 1930, together with the beetles could be mimicking a sympatric species of ant. Adult praying mantis mimicry of a wasp has been suggested for only one species based solely on its unique yellow and black coloration pattern, *Nemotha metallica* Westwood, 1845 (Stiewe & Shcherbakov, 2017). By far, ant mimicry in nymphs is the dominant pattern for hymenopteran mimicry in praying mantises, but adults mimicking wasps is barely present and certainly not studied.

Mantoididae is a relatively small family with only two described genera and 13 known extant species (Agudelo, 2014; Wieland & Svenson, 2018). The lineage is consistently recovered as the second extant branch of the praying mantis phylogeny, after Chaeteessidae (Svenson & Whiting, 2004, 2009; Wieland, 2013; Legendre et al., 2015; Rivera & Svenson, 2016; Svenson & Rodrigues, 2017), and is critical to understanding the early evolution of the order (Wieland, 2006, 2013; Klass, 1997; Klass & Meier, 2006). Other than the original publications describing species of Mantoididae, inclusion in some distributional records, and a focused study on the island distribution and natural history of one species (Wieland & Schütte, 2011), attention towards the family is limited. Agudelo, in his 2014 study describing *Paramantoida amazonica*, remarked that the genus *Mantoida* Newman, 1838 is in great need of systematic revision to address claims of undescribed diversity in the Neotropics. Expanding on this, six of the 11 extant species were described prior to 1900 (Ehrmann, 2002) with the next description occurring in the mid-1900s (Kaltenbach, 1957), followed much later by only three more species descriptions, including a fossil taxon (La Greca & Lombardo, 1990; Zompro, 2005). The most recently described species, *Mantoida toulgoeti* (Roy, 2010), was described from a single male specimen collected in French Guiana, though additional material has been subsequently located. Clearly, the early systematic work on the lineage that established a little more than half of the Mantoididae diversity coupled with the minimal attention over the past 100 years underscores a high potential for new discoveries within the family. Combining this with the widespread incidence of varying degrees of hymenopteran mimicry, the lineage represents a compelling ecological research system.

**Figure 1:**
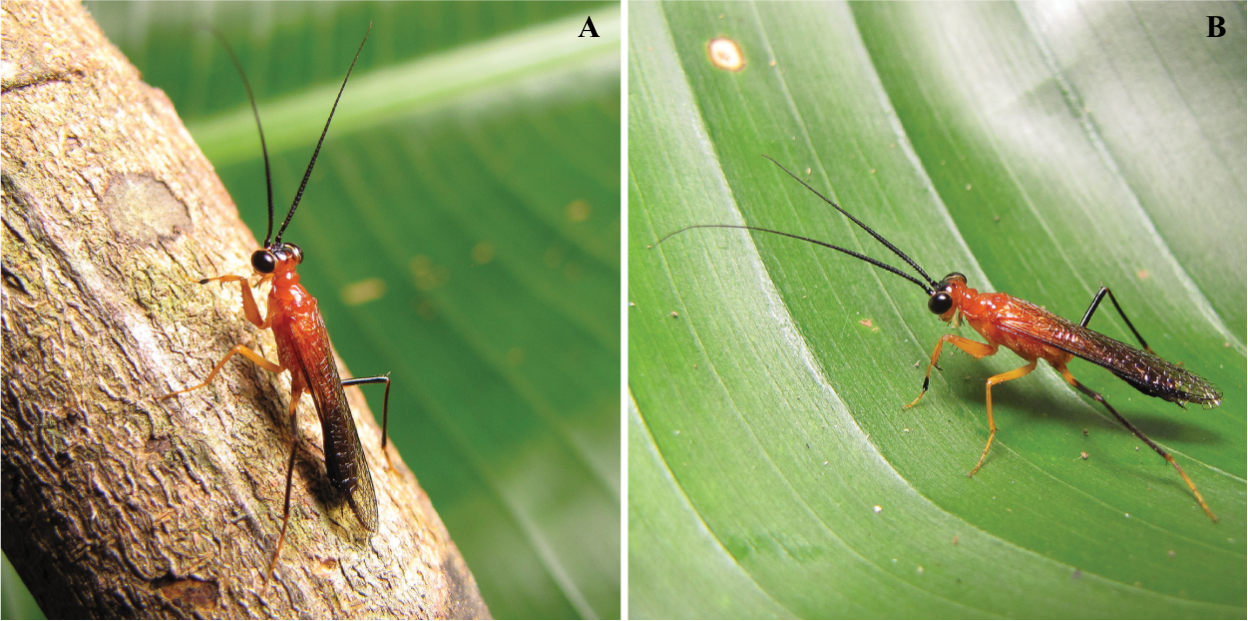
*Vespamantoida wherleyi* gen. nov. sp. nov. male holotype from Peru, live habitus photos (CMNHENT0129976). (A) On twig; (B) on leaf. Photo credit: Gavin J. Svenson.

**Figure 2:**
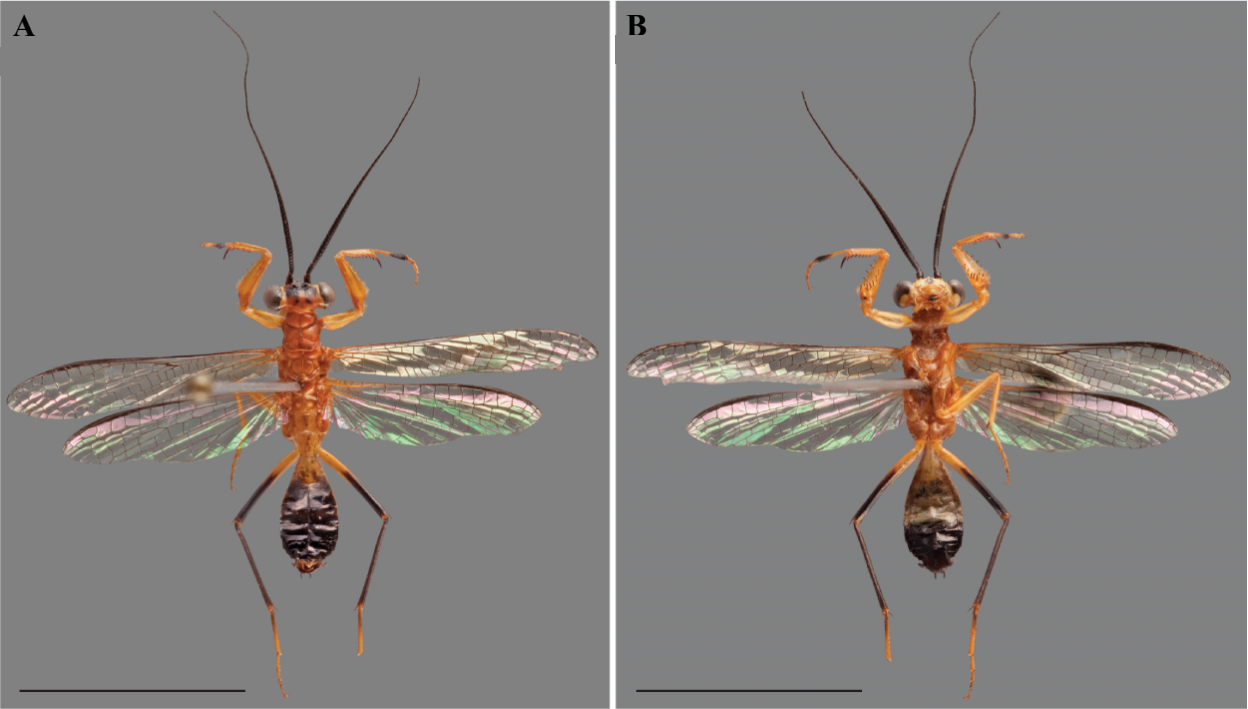
*Vespamantoida wherleyi* gen. nov. sp. nov. male holotype from Peru, specimen habitus photos (CMNHENT0129976). (A) Dorsal habitus; (B) ventral habitus. Scale bars = 1 cm. Photo credit: Rick Wherley.

In 2013, a highly unique specimen of praying mantis flew to a metal halide light trap that was positioned on the margins of a tributary to the Amazon River and dense primary rainforest to the south. The specimen was strikingly colored with bright red/orange contrasted with black markings on the head, eyes, and posterior half of the abdomen (Figs. 1 and 2). Part of the metathoracic legs, all of the mesothoracic legs, and the anterior raptorial legs were colored orange. No other known species of praying mantis is bright red/orange and black in coloration. Interestingly, this coloration pattern is extremely similar to the widespread black-orange-black color pattern present across many hymentoperan families (Mora & Hanson, 2019). The specimen’s morphology conformed to the general wasp-like shape seen in other Mantoididae and demonstrated wasp-like movement patterns and other behavioral similarities.

We hypothesize that this unique specimen among praying mantises represents a new species and, by extension, presents a novel pattern of conspicuous wasp mimicry. Therefore, the central goal of this study is to test if this specimen could be an aberrant color morph of a known species through careful comparison with the known diversity. To accomplish this, we utilize evidence from external morphology, dissected male genitalia, measurement data, geographic distribution records, and historical literature. We also provide, for the first time, digital recordings of behavioral evidence of hymenopteran mimicry in any species of praying mantis.

## Materials and Methods

Nocturnal field sampling using a tuned metal halide light system positioned above a vertically oriented sheet was conducted across eight nights (10–17 February) in 2013 at the Madre Selva Biological Research Station in the Loreto Province of Peru (GPS coordinates: −3.62096 −72.24744). Permission to conduct fieldwork and sample specimens of Mantodea was granted by the Peruvian Ministerio de Agricultura, Dirección General Forestal y de Fauna Silvestre (Resolución Directoral N° 006-2013-AG-DGFFS-DGEFFS). The weather was humid with intermittent rain across the sampling week. A single bright red/orange Mantoididae specimen arrived within an hour after sunset and no others were found throughout the sampling period. The specimen was placed in a live capture 50 ml vial and kept overnight. The specimen was released into a 500 cm by 500 cm by 1 m mesh cage with natural vegetation positioned within. After a few hours, the specimen was video recorded using a Canon SX10IS to capture brief movements. We have edited this video for content and duration to demonstrate locomotion and behavior.

A total of 142 specimens of *Mantoida* and *Paramantoida* were examined to compare directly with our newly sampled specimen from Peru (Table S1). Additional comparative morphological information, including genitalic illustrations, was sourced from Schwarz & Roy (2019), Agudelo (2014), Wieland (2013), Ehrmann (2002), Klass (1997), Terra (1995), La Greca & Lombardo (1990), and Giglio-Tos (1927). All examined material is deposited in the Cleveland Museum of Natural History, Cleveland, USA (CMNHENT), the Museum national d’Histoire naturelle, Paris, France (MNHN), or the National Museum of Natural History, Smithsonian Institution, Washington, DC, USA (USNM) (Table S1). For specimens without georeference data, we used Google Earth, Google Maps, or GeoNET to obtain the approximate coordinates, when possible. Specimens deposited in CMNHENT and USNM were databased and made available using protocols developed through the InvertEBase Thematic Collections Network and are available at https://hol.osu.edu/ as well as multiple online data aggregators. We used all georeferenced specimen records from our own database as well as some georeferenced records available in the literature (Agudelo, 2014) to construct a genus-level distribution map for 155 specimens of Mantoididae (Data S1). This map data can be downloaded as a KML file to be viewed in Google Earth (Data S2). It was impossible to verify all species level identifications for species of *Mantoida* present in the database and in our own collections, as the group is in need of taxonomic revision due to uncertainty of species boundaries and validity of names (Rehn, 1951; Agudelo, 2014). Therefore, we present nearly all specimen records under *Mantoida* without species determinations, with the exception of *Mantoida maya* Saussure & Zehntner, 1894 and *Mantoida brunneriana* Saussure, 1871. Based on findings in another early evolving Neotropical praying mantis lineage, Liturgusidae (Svenson, 2014), disjunct distribution patterns were found to be good indicators of species boundaries and most species did not have ranges extending across major biomes (e.g., the Amazon Basin).

Specimen examination was completed using a Leica M165C stereomicroscope. Dissecting procedures were conducted as described in Rodrigues & Cancello (2016) and morphological nomenclature follows Brannoch et al. (2017). Key morphological features were used to separate the newly collected specimen from other Mantoididae species, which include head, thorax, legs, and male genital structures (described in detail below). Coloration was used in the description, but relates to either contrasting color patterns (dark markings on a lighter background) or bright red/orange coloration. In the case of the observed red color, we reference the living specimen rather than the preserved holotype. Fading of the red/orange coloration has occurred in the preserved specimens and resulted in the loss of the bright color intensity. Our measurements followed a standard system established in Brannoch et al. (2017) where easily defined homologous landmarks are used. Measurements taken include: body length (center of ocellar tubercle to the abdominal tip), head length (clypeo-labral junction to the vertex), prothorax length (anterior to posterior margins along the midline) and width (lateral margin to margin at the widest point), metazone length (supracoxal sulcus to the posterior edge of the prothorax), forecoxa length (basal articulation to the base of the coxal lobes), forefemur length (proximal base to the distal terminus of the genicular lobe), foretibia length A (proximal bend to the tarsal insertion), foretibia length B (proximal bend to the terminus of the spur), meso- and metafemur length (from the articulation with the trochanter to the articulation with the tibia), meso- and metatibia length (from the articulation with the femur to the articulation with the tarsus), and forewing and hindwing length (convergence of the Analis veins to the distal terminus of the wing). All measurements are (range and average, when possible) given in millimeters (Data S3) and were collected using a Leica M165C stereomicroscope fitted with an IC80 HD coaxial video camera using the live measurement mode of the Leica Application Suite.

High-resolution images were taken with a Passport Storm© system (Visionary Digital™ 2012), with a Stackshot z-stepper, a Canon 5d SLR, MP-E 65 mm macro lens, three Speedlight 580EX II flash units. Z-stepper was controlled with Zerene stacker 1.04, and the images were stacked using the P-Max protocol. The initial image process was done with Adobe Lightroom 3.6, and further processed with Adobe Photoshop for minor background corrections and inclusion of scale bars. Illustrations were produced with Adobe Illustrator by tracing over high-resolution images of morphology. Plates were constructed with Adobe Photoshop and Illustrator.

The electronic version of this article in portable document format will represent a published work according to the International Commission on Zoological Nomenclature (ICZN), and hence the new names contained in the electronic version are effectively published under that Code from the electronic edition alone. This published work and the nomenclatural acts it contains have been registered in ZooBank, the online registration system for the ICZN. The ZooBank LSIDs (Life Science Identifiers) can be resolved and the associated information viewed through any standard web browser by appending the LSID to the prefix http://zoobank.org/. The LSID for this publication is: urn:lsid:zoobank.org:pub:1CB27FFD-35CF-4610-896C-3882833D5727. The online version of this work is archived and available from the following digital repositories: PeerJ, PubMed Central, and CLOCKSS.

## Results

**Figure 3:**
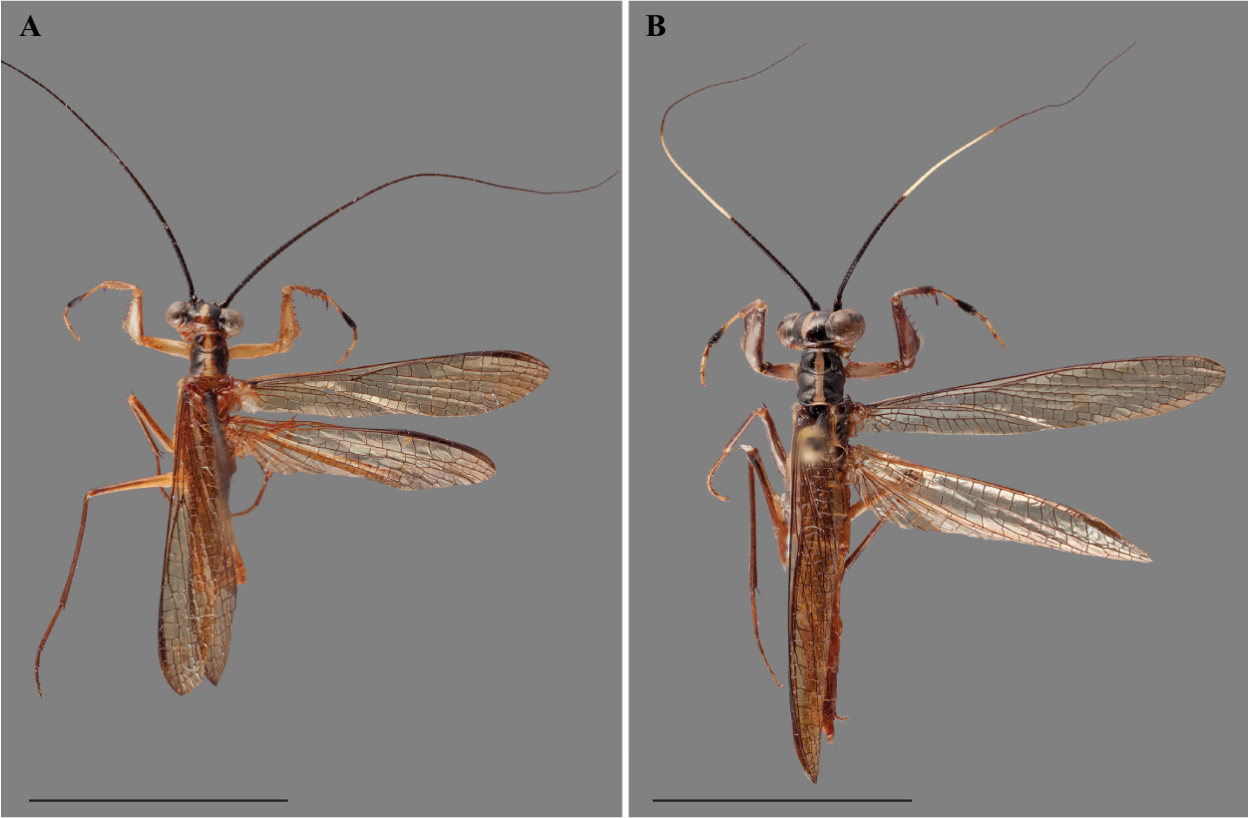
*Vespamantoida toulgoeti* from French Guiana, dorsal habitus photos. (A) Male (MNHN); (B) female (MNHN). Scale bars = 1 cm. Photo credit: Rick Wherley.

**Figure 4:**
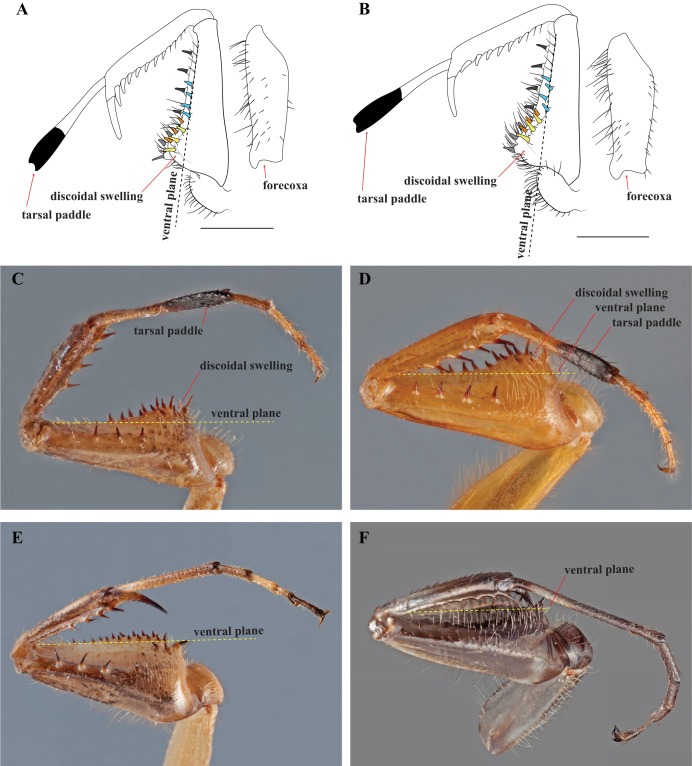
Prothoracic legs of Mantoididae genera illustrating diagnostic characters. (A) *V*. *toulgoeti*; (B) *V*. *wherleyi* gen. nov. sp. nov.: Illustrations highlighting tarsal paddle, discoidal swelling relative to the ventral plane, and the arrangement pattern of the anteroventral femoral spines (later row of three colored yellow, medial row of three colored orange, distally aligned row of four colored light blue). Images of prothoracic legs from the posterior perspective taken at an oblique angle demonstrate two synapomorphies for *Vespamantoida* including the tarsal paddle and the discoidal swelling. Dotted yellow lines indicates the surface of the ventral plane as established by alignment with the distal anterior margin of the forefemora. (C) *V*. *toulgoeti* (male, MNHN); (D) *V*. *wherleyi* gen. nov. sp. nov. (holotype male, CMNHENT0129976); (E) *Mantoid*a sp. (male, CMNHENT0131962); (F) *Paramantoida amazonica* (male, USNMENT01091953). Scale bars = 1 mm. Photo credits: Rick Wherley. Illustration credit: Henrique M. Rodrigues.

After examination of numerous specimens representing two determine species (*Mantoida maya* and *Mantoida brunneriana*), a number of morphospecies of *Mantoida*, and *Paramantoida amazonica*, we affirm that the Peruvian specimen clearly belonged within the family Mantoididae. This determination was based on the presence of the RA and RP+M wing vein spacing synapomorphy as well as conforming to the additional characters outlined for the family by Schwarz & Roy (2019), except for one discussed below. In addition, the Peruvian specimen was clearly not allied with *Paramantoida amazonica* based on the presence of an additional two posteroventral spines on the forefemora. Further, it appeared to diverge significantly from all examined specimens of *Mantoida* except for those determined as *Mantoida toulgoeti* (Fig. 3). Both the Peruvian specimen and *Mantoida toulgoeti* retain two obvious synapomorphies on their forelegs that differentiate them from all other examined species of *Mantoida*, as well as *Paramantoida*. First, the discoidal spines are raised above the ventral plane of the femur on a “discoidal swelling” that elevates the base of the spines well above the ventral plane of the femora (Figs. 4A–4D). Second, the distal half of the first segment of the foretarsi is flattened and laterally expanded while also being colored black, forming a “tarsal paddle” (Figs. 4A–4D). Both of these character states are absent in *Paramantoida* and the specimens of *Mantoida* that we observed (Table S1), which have a flat ventral plane of the forefemora and straight, unaltered foretarsal segments (Figs. 4E and 4F). However, it has been suggested that the discoidal swelling might be present in certain species of *Mantoida*, namely *Mantoida luteola* (E. Shcherbakov, 2019, personal communication) and possibly *Mantoida beieri* based on the illustration (see Fig. b in Kaltenbach, 1957). We did not have access to determined specimens of these species to confirm, but it does indicate that there is potential overlap in the presence or degree of presence of a discoidal swelling.

**Figure 5:**
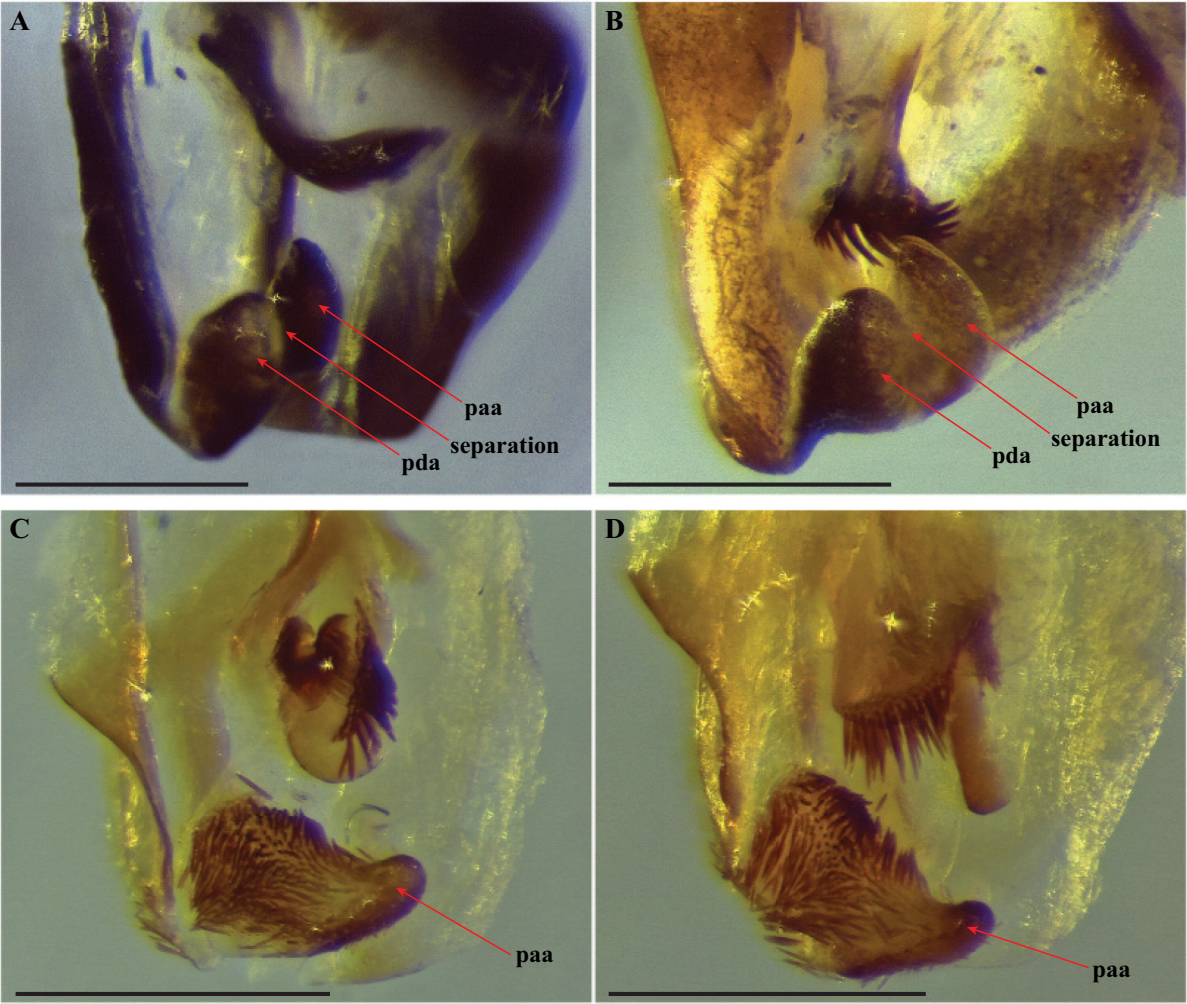
Terminus of the left phallomere complex of male genitalia. The composition of the distal processes that is comprised of the pda (posterior ventral process) and the paa (posterior process) are indicated as clearly developed and fused, with a medial suture and a bifurcate terminus in: (A) *Paramantoida amazonica* (USNMENT01091953); and (B) *Mantoida* sp. (CMNHENT0131933). For *Vespamantoida*, the pda appears to be highly reduced or absent, the distal process comprised entirely of the paa: (C) *V*. *toulgoeti* (CMNHENT0130060); and (D) *V*. *wherleyi* gen. nov. sp. nov. (CMNHENT0129976). Scale bars = 0.5 mm. Photo credit: Gavin J. Svenson.

**Figure 6:**
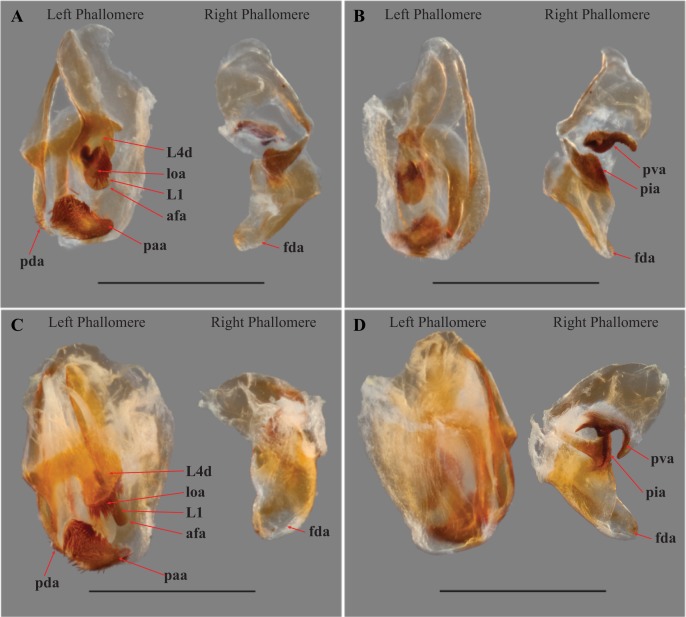
Male genital complex of *Vespamantoida* species. *V*. *toulgoeti* (CMNHENT0130060): (A) dorsal view; (B) ventral view. *V*. *wherleyi* gen. nov. sp. nov. (CMNHENT0129976): (C) dorsal view; (D) ventral view. afa, anterior apodeme; L1, principle sclerite; L4d, dorsal extension of L4B; loa, membranous lobe; paa, posterior process; pda, distal process; pva, posterior ventral process (right phallomere); pia, process posterolateral to pva (right phallomere); fda, main posterior lobe. Scale bar = 1 mm. Photo credit: Henrique M. Rodrigues.

Our extensive examination of male genitalia incorporated direct observation of dissected morphology from *Paramantoida amazonica* (Fig. 5A), six morphospecies of *Mantoida* (Fig. 5B) from Nicaragua, Bolivia, French Guiana, and Peru (CMNHENT0131924, -933, -935, -992, -995, and -997; Table S1), *Mantoida toulgoeti* (Fig. 5C), and the Peruvian specimen (Fig. 5D). We also examined published images and illustrations of *Mantoida brunneriana* (see Fig. 2 in La Greca & Lombardo, 1990; see Fig. 9d in Agudelo, 2014), *Mantoida argentinae* La Greca & Lombardo, 1990 (see Fig. 6 in La Greca & Lombardo, 1990), *Mantoida ronderosi* La Greca & Lombardo, 1990 (see Fig. 8 in La Greca & Lombardo, 1990), *Mantoida luteola* Westwood, 1889 (see Fig. 9c in Agudelo, 2014), and *Mantoida schraderi* Rehn, 1951 (see Fig. 44-45 in Klass, 1997). This taxonomic coverage captured at least 11 of the 13 described species in the family. These examinations confirmed the divergence of both *Mantoida toulgoeti* (Fig. 5C) and the Peruvian specimen (Fig. 5D) in the following three ways:

1. The presence of dense setae on the distal process of the ventral phallomere is similar to the *Mantoida* species-group established by Agudelo (2014) that includes the smaller sized, setae bearing taxa *Mantoida tenuis*, *Mantoida luteola*, *Mantoida maya*, and *M. ronderosi*. However, the presence of such setae clearly separates them from *Paramantoida* (Fig. 5A) and the second species-group established by Agudelo (2014) that includes *Mantoida nitida*, *Mantoida argentinae*, *Mantoida brunneriana*, *Mantoida fulgidipennis*, and *Mantoida schraderi*. None of these six species, including the type species of *Mantoida* (*Mantoida nitida*), bear any setae on the distal process of the ventral phallomere (Fig. 5B).
2. The distal process of the ventral phallomere is comprised entirely of an enlarged, conical paa that is densely pilose and concave on the dorsal surface in the basal half; the pda is highly reduced or absent (Figs. 5C, 5D and 6). In all other examined specimens of Mantoididae, including *Paramantoida amazonica* (Fig. 5A), six morphospecies of Agudelo’s non-setose *Mantoida* species (Fig. 5B), and the illustration and image of Agudelo’s setae bearing *Mantoida* species including *Mantoida ronderosi* (see Fig. 8 in La Greca & Lombardo, 1990) and *Mantoida luteola* (see Fig. 9c in Agudelo, 2014), there was conformation with the established synapomorphy for Mantoididae of a distal process being comprised of a fused pda and paa (Schwarz & Roy, 2019). This fusion is easy to see in Mantoididae species due to the membranous medial line between the pda and paa (Figs. 5A and 5B). In addition, the process typically terminates in a truncate or bifurcate shape that preserves evidence of the compound origins of the process. It has been suggested that the pda can be smaller or reduced compared to the paa (La Greca & Lombardo, 1990), but it has never been observed to be highly reduced or nearly absent in the family. This suggests a re-description of Mantoididae will be necessary to include this new character state.
3. The composition of the dorsal lamina of L4d (component part of L4B, see Klass, 1997) and the phalloid apophysis (afa) resemble that of *Mantoida ronderosi*, which were described as being highly unique compared to all other *Mantoida* species by La Greca & Lombardo (1990), possibly not belonging within the genus. In *Mantoida toulgoeti* and the Peruvian specimen (Fig. 6), the L4d projects posteriorly over the L1 and afa and terminates with a broadly rounded and sclerotized loa (see Fig. 44–45 in Klass, 1997) that is fringed with a row of densely packed, elongated and robust setae (Fig. 6). The L1 forms a tight crevice anterior and under the loa, which is highly sclerotized, then projects posteriorly out from under the loa to form a smooth, sclerotized structure. The afa is not significantly developed beyond the termination of the distal end of L1. By comparison, the L1 and afa in *Mantoida ronderosi* and *Mantoida luteola* fails to project beyond the loa and forms a more robustly sclerotized, complex, and setose afa (see Fig. 8 in La Greca & Lombardo, 1990; see Fig. 9c in Agudelo, 2014).

To resolve the discrepancy in both external and male genital characters between *Mantoida toulgoeti* and the Peruvian specimen with all other examined specimens of *Mantoida* and *Paramantoida*, we erect a new genus of Mantoididae, *Vespamantoida* gen. nov. We transfer *Mantoida toulgoeti* to this new genus and describe the Peruvian specimen as a new species. These actions are supported by a number of external morphological characters, distinct characters from the male genital complex, measurement data, unique patterns of distribution, and information present in the literature from *Mantoida* and *Paramantoida*.

*Vespamantoida* gen. nov. urn:lsid:zoobank.org:act:16B451EE-222D-45EA-A34E-570D7DAA0B6E

Type-species—*Vespamantoida wherleyi* sp. nov. by original designation

Diagnosis—Forefemur narrow basally, only slightly wider than apex; discoidal spines elevated well above the ventral plane on a pronounced discoidal swelling (Figs. 4A–4D). Anteroventral femoral spines loosely arranged in two rows of three spines followed by a single row of four spines (Figs. 4A and 4B). Distal half of the first segment of the foretarsi flattened, laterally expanded, and black, forming a tarsal paddle (Figs. 4A–4D). Distal process of the ventral phallomere densely setose from narrowly rounded terminus to a heavily sclerotized, broad base; process comprised entirely of the paa, the distal process (pda) is highly reduced or absent (Figs. 5C and 5D).

Distribution—Although species of *Mantoida* are recorded across a broad range from Florida, USA to Northern Argentina, both *Paramantoida amazonica* and both species of *Vespamantoida* are restricted. *Paramantoida amazonica* is only recorded from a long transect that roughly follows the northern boundary of the Rio Negro from Manaus to the Parque Nacional Serrania La Neblina in southern Venezuela. The two species of *Vespamantoida* are found far from each other on near opposite sides of the Amazon Basin. This extremely disjunct distribution is supportive of the distinct boundary between the two species.

**Figure 7:**
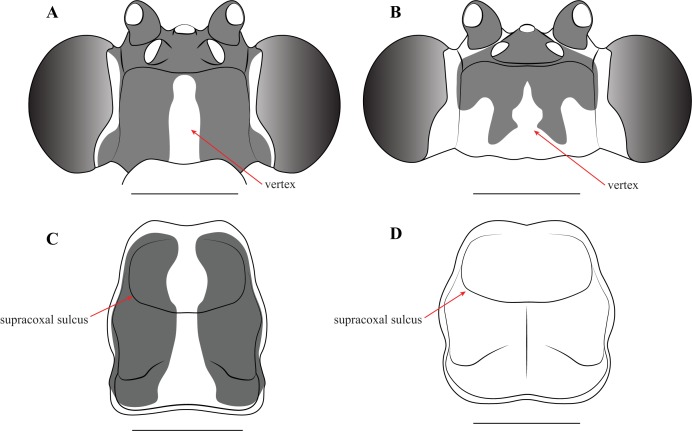
Illustrations of diagnostic morphology and coloration patterns of the head and prothorax of *Vespamantoida* males. Head from dorsal perspective: (A) *V*. *toulgoeti*; (B) *V*. *wherleyi* gen. nov. sp. nov. Prothorax from dorsal perspective: (C) *V*. *toulgoeti*; (D) *V*. *wherleyi* gen. nov. sp. nov. Scale bar = 1 mm. Illustration credit: Henrique M. Rodrigues.

Description—Small sized insects, body coloration ranging from reddish brown to bright red/orange and black in live specimens (Figs. 1–3). Vertex as long as wide (Figs. 7A and 7B). Eyes globous in dorsal view (Figs. 7A and 7B) and reniform in frontal view. Ocelli medium sized, rounded, evenly spaced from each other; ocellar plate black. Antennae as long as the body in both sexes (as observed in *V*. *toulgoeti*). Lower frons wider than high. Prothorax as long as wide, supracoxal sulcus deep, supracoxal dilation weakly pronounced (Figs. 7C and 7D). Prozone almost as long as metazone. Forecoxae short and robust, with several long setae on the anterior margin and few short setae on the posterior surface, coxal apical lobes reduced and divergent (Figs. 4A and 4B). Forefemur short and robust; approximately two times wider at the base than at the apex, moderately expanded laterally; posterior surface bearing setae; *F* = 4PvS/3DS/10AvS; first discoidal spine larger than the others and located on a discoidal swelling; the first six proximal anteroventral spines loosely arranged in two parallel rows, the four distal spines loosely arranged in a single row, after that, long bristles are located on the anteroventral edge; femoral brush on the anterior surface located above anteroventral edge that bears sparse setae; genicular spines absent (Figs. 4A–4D). Foretibiae with *T* = 4PvS/7AvS and three to four robust setae on the base of the anteroventral edge (Figs. 4A and 4B). First segment of the foretarsi flattened, laterally expanded, and black, forming a tarsal paddle (Figs. 4A–4D). Forewings hyaline or translucent, well-developed (Figs. 1–3). Hindwings also hyaline or translucent, anal area reduced, no longer than two-thirds of the discoidal area length (Figs. 1–3). Meso- and metathoracic legs with elongate genicular and apical spurs. Mesofemora longer than mesotibiae and metafemora shorter than metatibiae. Anterior two segments of the abdomen constricted in males, forming a narrowed wasp waist (Figs. 2 and 3A); females with gradually tapering abdomen (Fig. 3B).

Male genitalia (Fig. 6): The left phallomere is elongated, forming a rectangular shape. The distal process of the ventral phallomere is comprised entirely of an enlarged, conical paa of the L2 sclerite that is densely pilose and concave on the dorsal surface in the basal half. The setae on the paa is oriented in a semi-whorl with a central point in the basal three quarters; the anterior setae oriented anteriorly, posterior setae oriented posteriorly. The terminus of the paa without setae and tapering to a small, rounded point. The pda of L4A is highly reduced (Fig. 6). The L4d projects posteriorly over the L1 and afa and terminates with a broadly rounded and sclerotized loa (see Fig. 44-45 in Klass, 1997) that is fringed with a row of densely packed, elongated and robust setae (Fig. 6). The L1 forms a tight crevice anterior and under the loa, which is highly sclerotized, then projects posteriorly out from under the loa to form a smooth, sclerotized structure. The afa is not significantly developed beyond the termination of the distal end of L1. The L1 can be either elongated and narrow, projecting posteriorly well beyond the loa or it can be broad, rounded and shaped more like a paddle, not projecting much beyond the loa. The L4A weekly sclerotized with an evenly truncate, distal terminus. The main posterior lobe (fda) of the right phallomere is elongated, tapering to a membranous, rounded terminus. The pia is large and triangular with the externally facing margin highly sclerotized, appearing like a shark tooth, the terminus of which is the internal point of the pia; the pva is S-shaped or C-shaped with a highly sclerotized region on the distal end or centrally, projecting out from the C.

Etymology—*Vespamantoida* from the Latin word *vespa*, meaning “wasp,” and *Mantoida*, the name of the type genus of the family Mantoididae. This is a reference to the striking similarity between the type species and wasps.

*Vespamantoida toulgoeti* (Roy, 2010)
*Mantoida toulgoeti* (Roy 2010; Agudelo 2014; Roy 2019)

Type locality—French Guiana, route Régina pk 62, degrad Corrèze

Diagnosis—Head and thorax black dorsally with a pale medial band, remaining thorax and abdomen a light brown to rust color in males, darker color with medial band continuing from pronotum to the metathorax in females; no central keel on the pronotum. Legs pale to brown with the metathorcic tibiae darkened to a medium brown color in males, darker in females. Frontal sclerite evenly colored with pale brown. Flagellum of antennae of the male uniformly slender. The L1 of the male genital complex forming a broad paddle that orients with the afa to form a small pocketed space (Figs. 3, 4A, 4C, 5C, 6A, 6B, 7A and 7C).

**Figure 8:**
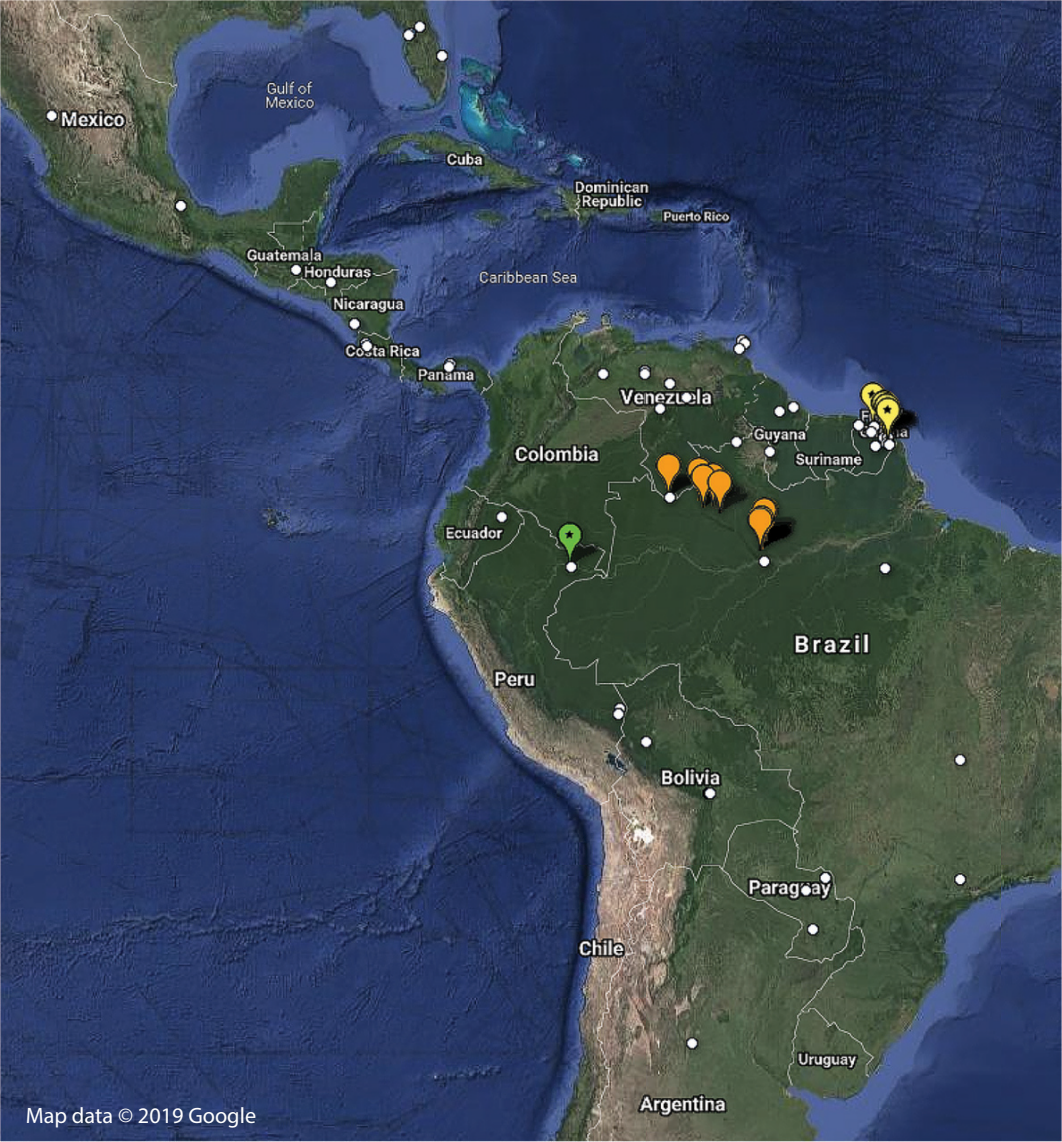
Distribution of examined georeferenced specimens of Mantoididae (see Table S1; Data S1). Small white circles indicate specimens of *Mantoida*, which range from Northern Argentina to Mexico and Florida, USA. Large orange markers indicate specimen localities for *Paramantoida amazonica* in Northern Brazil and the southern border of Venezuela. Yellow markers with a star indicate localities for *Vespamantoida toulgoeti*, known only from French Guiana. The single green marker with a star is the type locality of *Vespamantoida wherleyi* gen. nov. sp. nov. from Northern Peru. Map data © 2019 Google.

Distribution—Only known from Northern French Guiana through a number of independent sampling expeditions to the region (Fig. 8; Table S1).

Redescription—Vertex black with a light, anterior/posterior oriented medial band (Fig. 7A); ocelli slightly reduced on the female compared to male; antennae gently tapering towards the terminus, transitioning from black to dark brown on males; black at the base, light colored medially, encompassing about 15 antennomeres, then colored dark brown to the distal terminus in females (Fig. 3). Lower frons pale in color, without contrasting markings. Terminus of maxillary palpi black. Prothorax black with a light, anterior/posterior oriented medial band (Fig. 7C). The dark coloration extends into the mesothorax with the light colored medial band in females; meso- and metathorax in males is pale brown or rusty in coloration. Dorsal and ventral surface of the abdomen pale brown or rusty in males and females. Hings in males and females with brown veination, the cell membranes pigmented with a slightly translucent smoke. The costal area of the forewing pigmented light brown proximally, dark brown in the distal third of the wing; the anterior veinal margin (avm) pigmented pale proximally, then darkening distally; the posterior subcostal vein (ScP) pigmented nearly black proximally and dark brown distally to the terminus. The pro-, meso-, and metathoracic legs of males and females pigmented light brown with darker, unmottled coloration on the femora and especially the tibiae. The first segment of the foretarsi light brown, then forming a black tarsal paddle; the subtending tarsal segments light brown and cylindrical (Figs. 4A and 4C). Forefemora with sparse, long setae in the region on the posterior side of the discoidal spines. Subgenital plate of the female light colored.

Male genitalia (Figs. 6A and 6B): The L4A of the left phallomere complex elongated, rectangle. The L4d projects medially and is bulbous and rounded with a dense row of long, robust setae on distal projection (loa). The crevice of the L1 proximal and ventral to the loa is fringed with fine setae and highly sclerotized, forming a C-shape. The L1 sclerite forming a broad, flattened paddle that projects just beyond the loa marginal setae; weekly sclerotized and circular in shape. The combination of the L4d/loa projection and the L1 forming a small pocketed space. The right phallomere with a pia forming triangle, shaped like a shark tooth, but curved with the terminus oriented internally; the pva is S-shaped with a highly sclerotized region on the distal end; the surface with small, densely spaced, sharp tubercles.

Measurements—♂ (*n* = 5) Body length: 12.02–12.7 (12.33); Head length: 1.63–1.92 (1.8); Prothorax length: 1.61–1.76 (1.68); Prothorax width: 1.52–1.6 (1.56); Metazone length: 0.87–0.96 (0.93); Forecoxa length: 1.72–1.93 (1.86); Forefemur length: 2.25–2.43 (2.34); Foretibia length A: 1.26–1.42 (1.36); Foretibia length B: 1.92–2.04 (1.98); Mesofemur length: 2.68–2.76 (2.73); Mesotibia length: 2.46–2.65 (2.59); Metafemur length: 3.98–4.16 (4.04); Metatibia length: 4.47–4.76 (4.59); Forewing length: 11.51–12.06 (11.83); Hindwing length: 10.07–10.56 (10.31).

♀ (*n* = 1) Body length: 16.33; Head length: 2.27; Prothorax length: 2.13; Prothorax width: 1.96; Metazone length: 1.1; Forecoxa length: 2.28; Forefemur length: 2.68; Foretibia length A: 1.64; Foretibia length B: 2.26; Mesofemur length: 3.06; Mesotibia length: 2.91; Metafemur length: 4.81; Metatibia length: 5.41; Forewing length: 14.02; Hindwing length: 12.45.

*Vespamantoida wherleyi* sp. nov.
urn:lsid:zoobank.org:act:6B2F0EC1-54DC-4DAB-9E68-52583C52B344

Holotype—Male, Peru: Loreto Province, Madre Selva Biological Research Station, −3.62096, −72.24744, 10–17 February 2013, Coll: G.J. Svenson, Tissue sample #019 (Repository: Cleveland Museum of Natural History—CMNHENT0129976).

Type Locality—Peru, Loreto Province, Madre Selva Biological Research Station.

Diagnosis—Head is bright red/orange with symmetrical black markings on the anterior half of the vertex and the ocellar tubercle; the pronotum, thorax, and first three segments of the abdomen bright red/orange. The forelegs and mesothoracic legs orange. The metathoracic legs are orange in the proximal half of the femur, then black to reddish to the terminus of the tibiae; the tarsi are orange. The flagellum of the antennae of the male thickening from base to the broadest antennomers 7–9, then tapering thinner to the terminus. A dark black spot centrally located on the frontal sclerite. The L1 shaped as an elongated bar with a slight curve distally that orients towards the afa, to form a claw-like structure with a broadly open gap (Figs. 1, 2, 4B, 4D, 5D, 6C, 6D, 7B and 7D).

Comments—This species is distinct from *V. toulgoeti* in the coloration of the specimen, the shape of the antennae and the male genitalia. The distribution of the two species, *V. toulgoeti* only known from French Guiana and *V. wherleyi* only from Northern Peru, allows for easy diagnosis between the congeneric species. The female of *V*. *wherleyi* is currently unknown.

Distribution—Known only from the type locality on the southern bank of the Amazon River in Northern Peru (Fig. 8; Table S1). Sympatric species of *Mantoida* were sampled from the same location, but none exhibited coloration outside of dark brown and black that is typical for the genus.

Description—Vertex bright red/orange with black, symmetrical markings on the anterior half that extend to the ocellar tubercle (Fig. 7B); flagellum of the antennae of the male thickening from base to the broadest antennomers 7–9, then tapering thinner to the terminus (Fig. 2); flagellum uniformly black. Lower frons pale in color, but with a distinct centrally located black spot. Terminus of maxillary palpi black. Prothorax, meso- and metathorax uniformly bright red/orange (Fig. 7D). Anterior three segments of the abdomen orange, the second and third forming the narrowed wasp waist; all posterior abdominal segments are black (Fig. 2). Wings with mostly brown pigmented veins, but all veins in the proximal tenth are pigmented orange; cell membranes are slightly translucent to clear. The costal area of the forewing pigmented light orange proximally, dark brown in the distal third of the wing; the avm pigmented orange at the base, then darkening to brown distally; the posterior ScP pigmented orange at the base, then black to the terminus. The pro- and mesothoracic legs of pigmented orange. The proximal half of the femur and tarsi in the metathoracic legs are pigmented orange, the distal half of the femur and tibiae are black. The first segment of the foretarsi light brown, then forming a black tarsal paddle; the subtending tarsal segments light brown and cylindrical (Figs. 4B and 4D). Forefemora with dense, long setae in the region to the proximal side of the discoidal spines.

Male genitalia (Figs. 6C and 6D): The L4A of the left phallomere complex squat, nearly square-shaped. The enlarged L4d sclerite bulbous and rounded with a dense row of long, robust setae on distal margin (loa). The L1 forming a small, highly sclerotized V-shaped turn proximal and ventral to the L4d projection; fringed with dense, very short setae. The L1 sclerite projecting under and beyond the distal margin of the loa and shaped as an elongated bar with a slight curve distally that orients towards the loa, slightly sclerotized along the length and without setae. The L1 and loa form a claw-like structure with a broadly open gap when viewed in profile, unlike the small space formed by the more truncated structure present in *V. toulgoeti*. The right phallomere with the pia forming triangle shaped like a shark tooth, projecting straight with a highly sclerotized upper or external margin; the pva is C-shaped with a highly sclerotized central projection; the surface of the pia and pva with small, densely spaced, sharp tubercles.

Measurements—♂ (*n* = 1) Body length: 12.45; Head length: 1.83; Prothorax length: 1.65; Prothorax width: 1.63; Metazone length: 0.89; Forecoxa length: 1.84; Forefemur length: 2.33; Foretibia length A: 1.51; Foretibia length B: 1.96; Mesofemur length: 2.70; Mesotibia length: 2.49; Metafemur length: 3.92; Metatibia length: 4.23; Forewing length: 11.38; Hindwing length: 9.68.

Etymology—Named for Rick Wherley, a valued member of the systematic entomology group at Cleveland Museum of Natural History. He has enriched the scientific content of the Museum for many years through imaging and computational improvements.

Natural history—The type specimen was collected in dense, tropical rainforest near the banks of small tributary of the Amazon River in Northern Peru. The specimen flew to our sampling lights within an hour after dark and was taken from the sheet shortly after. No observations were made of the mantis in situ for fear it would fly away and not be found again. The specimen was placed in a small enclosure on natural vegetation taken from the surrounding habitat for observation. Short video clips were recorded of the mantis walking on a blunt stick within this placed vegetation (Video S1). The 19 second video, including two behavioral segments, clearly demonstrates a number of behavioral characteristics that are strikingly similar to the movement patterns of many hymenopteran wasps. First, the mantis walks forward, with the head in a lowered position, and rapidly sweeps side to side, rotating its body in an alternating, circuitous pattern as it moves forward. This pattern is broken up by larger redirections on the terminus of the stick in what appears to be a search behavior. A second similarity is the antennal movements beginning a rapid up and down pattern that appears to be contacting the substrate to feel or sense the environment while walking, then moving with a declining amplitude following a pause in walking. A third similarity is the slight up and down movement of the abdomen while walking that resembles a hymenopteran-like pumping or venting behavior. The fourth similarity is the rapidity of the mantis’ walking and the rapid start and stop to this motion.

## Discussion

We present multiple lines of evidence from external morphology (Figs. 4 and 7), genital dissections (Figs. 5 and 6), and geographic distribution (Fig. 8) that support the creation of a third genus within Mantoididae, the re-assignment of *Mantoida toulgoeti* to this new genus, and the description of the new species. *V. wherleyi* was described based on clear morphological boundaries with the congeneric *V. toulgoeti* that include striking differences in external coloration (Figs. 2 and 3), multiple male genital characters in the left phallomere complex (Fig. 6), and an extremely disjunct distribution (Fig. 8). Therefore, we found that the unique specimen from Peru was not an aberrant color morph of a known species based on these additional lines of evidence that outline its status as a distinct species. In addition, we conclude that *V. wherleyi* represents a novel pattern of wasp mimicry in all praying mantises, one that exploits conspicuous coloration in adults. We also confirmed prior notions of revisionary need within the genus *Mantoida*. Although the new taxon we describe herein is clearly divergent, some of the characteristics have similarities with species within *Mantoida*. Some of these species, namely those bearing setae on the distal process of the left phallomere, are likely divergent enough to require their own genus-group.

The most profound aspect of the discovery of *V. wherleyi* is the high degree of similarity it has, both morphologically and behaviorally, with hymenopteran wasps. Although a number of non-mantoididae praying mantis taxa have been documented to exhibit some degrees of ant mimicry in nymphal stages, and more rarely, in wingless adult females (Milledge, 1990; Agudelo & Rafael, 2014), only one enigmatic species from Southeast Asia, *N. metallica*, has been suspected of exhibiting wasp mimicry based on its unique color pattern (Stiewe & Shcherbakov, 2017). The Mantoididae family, on the other hand, includes both nymph and winged adult mimics of hymenopterans (ants and wasps, respectively), which includes a generalized hymenopteran body shape and the presence of a wasp waist (Jackson & Drummond, 1974; Deyrup, 1986; Agudelo, 2014). However, none of the previously described diversity in the family go so far as *V. wherleyi* in its presentation of bright red/orange coloration that is contrasted with black markings. Exceedingly few adult praying mantises exhibit any bright, conspicuous coloration since camouflage is a centerpiece of mantodean ecological strategy (Edmunds, 1972, 1976; Svenson & Whiting, 2009; Svenson et al., 2016; Svenson, Medellin & Sarmiento, 2016).

Aposematism and mimicry are possibly the biological functions of the red/orange and black coloration of *V. wherleyi*, as this type of color pattern acts as warning coloration in other insect orders (María Arenas, Walter & Stevens, 2015) and is exhibited by some hymenopteran mimics (Mora & Hanson, 2019). Behaviorally, our novel video recordings clearly capture wasp mimicry that is reflected in short bursts of locomotory movement combined with abdominal and antennal motion that elicits clear connections with pompilid or ichneumonid wasps (Video S1). The diversity of wasps that exhibit such behavior is vast and we presume most or all of the Mantoididae species exhibit this behavioral mimicry in the adult stage, but their Earth tone coloration indicates a more generalized shape and movement mimicry of wasps. However, *V. wherleyi*, appears to have honed in on a more specialized coloration strategy that is found in 23 families of Hymenoptera (Mora & Hanson, 2019). Again, Pompilidae or Ichneumonidae wasps are prime candidates for a possible Batesian model, but a specific insect survey in the region would be necessary to conclude a model species.

## Conclusions

We present the first definitively documented species of an adult praying mantis mimicking, in coloration, behavior, and morphology, a conspicuously colored wasp. For a lineage of insect that largely relies on camouflage to avoid predation as adults, *V. wherleyi* has departed significantly from the normal vegetative or simple brown-green coloration patterns. The fitness gain by expressing bright red/orange coloration must be biologically relevant to *V. wherleyi*, but why only one brightly colored species in a lineage known to mimic Hymenoptera remains an open question.

## Supplemental Information

10.7717/peerj.7886/supp-1 Supplemental Information 1 Georeferenced specimens. Coordinates are standardized, database specimen codes are provided when present, locality is provided, and indicator marker format is presented.

10.7717/peerj.7886/supp-2 Supplemental Information 2 Google Earth KML file. Import and open in Google Earth to view distribution map presented in Fig. 8.

10.7717/peerj.7886/supp-3 Supplemental Information 3 Individual measurement data recorded for *Vespamantoida* specimens. All measurements are given in millimeters and based on the descriptions provided in the methods.

10.7717/peerj.7886/supp-4 Supplemental Information 4 List of the examined specimens of *Mantoida*, *Paramantoida*, and *Vespamantoida* gen. nov. Verbatim locality data provided for each specimen from label data. Institution of deposition abbreviations appear as the prefix to the database code included (when present), which are Cleveland Museum of Natural History (CMNH), Muséum national d’Histoire naturelle (MNHN), and National Museum of Natural History (USNM).

10.7717/peerj.7886/supp-5 Supplemental Information 5 Video of locomotory behavior of *Vespamantoida wherleyi* gen. nov. sp. nov. Short bursts of locomotory movement coupled with constant moving of the antennae and up-down movements of the abdomen. Video credit: Gavin J. Svenson.

## Additional Information and Declarations

### Field Study Permissions

The following information was supplied relating to field study approvals (i.e., approving body and any reference numbers): Permission to conduct fieldwork and sample specimens of Mantodea was granted by the Peruvian Ministerio de Agricultura, Dirección General Forestal y de Fauna Silvestre (Resolución Directoral N 006-2013-AG-DGFFS-DGEFFS).

### Data Availability

The following information was supplied regarding data availability: Individual measurement data recorded for Vespamantoida specimens are available in Data S3. All measurements are given in millimeters. Specimens measured include a single holotype male of V. wherleyi (repository Cleveland Museum of Natural History, specimen number CMNHENT0129976), five males of *V. toulgoeti* (repository Muséum national d’histoire naturelle (no accession numbers associated with three male specimens from MNHN), CMNHENT0130060, and CMNHENT0130146), and a single female of *V. toulgoeti* (repository Muséum national d’histoire naturelle, no accession number associated with specimen). A list of examined specimens is available as Table S1.

### New Species Registration

The following information was supplied regarding the registration of a newly described species: Publication LSID: urn:lsid:zoobank.org:pub:1CB27FFD-35CF-4610-896C-3882833D5727. Vespamantoida LSID: urn:lsid:zoobank.org:act:16B451EE-222D-45EA-A34E-570D7DAA0B6E. Vespamantoida wherleyi gen. nov. sp. nov. LSID: urn:lsid:zoobank.org:act:6B2F0EC1-54DC-4DAB-9E68-52583C52B344.
